# Tracking of epigenetic changes during hematopoietic differentiation of induced pluripotent stem cells

**DOI:** 10.1186/s13148-019-0617-1

**Published:** 2019-02-04

**Authors:** Olivia Cypris, Joana Frobel, Shivam Rai, Julia Franzen, Stephanie Sontag, Roman Goetzke, Marcelo A. Szymanski de Toledo, Martin Zenke, Wolfgang Wagner

**Affiliations:** 1 Helmholtz-Institute for Biomedical Engineering, Stem Cell Biology and Cellular Engineering, RWTH Aachen University Medical School, Pauwelsstraße 20, 52074 Aachen, Germany; 2 Institute for Biomedical Engineering – Cell Biology, RWTH Aachen University Medical School, Aachen, Germany; 3 Helmholtz-Institute for Biomedical Engineering, RWTH Aachen University, Aachen, Germany

**Keywords:** DNA methylation, Epigenetic, Hematopoiesis, Induced pluripotent stem cells, Mesenchymal stromal cells, Stromal support, Hematopoietic differentiation

## Abstract

**Background:**

Differentiation of induced pluripotent stem cells (iPSCs) toward hematopoietic progenitor cells (HPCs) raises high hopes for disease modeling, drug screening, and cellular therapy. Various differentiation protocols have been established to generate iPSC-derived HPCs (iHPCs) that resemble their primary counterparts in morphology and immunophenotype, whereas a systematic epigenetic comparison was yet elusive.

**Results:**

In this study, we compared genome-wide DNA methylation (DNAm) patterns of iHPCs with various different hematopoietic subsets. After 20 days of in vitro differentiation, cells revealed typical hematopoietic morphology, CD45 expression, and colony-forming unit (CFU) potential. DNAm changes were particularly observed in genes that are associated with hematopoietic differentiation. On the other hand, the epigenetic profiles of iHPCs remained overall distinct from natural HPCs. Furthermore, we analyzed if additional co-culture for 2 weeks with syngenic primary mesenchymal stromal cells (MSCs) or iPSC-derived MSCs (iMSCs) further supports epigenetic maturation toward the hematopoietic lineage. Proliferation of iHPCs and maintenance of CFU potential was enhanced upon co-culture. However, DNAm profiles support the notion that additional culture expansion with stromal support did not increase epigenetic maturation of iHPCs toward natural HPCs.

**Conclusion:**

Differentiation of iPSCs toward the hematopoietic lineage remains epigenetically incomplete. These results substantiate the need to elaborate advanced differentiation regimen while DNAm profiles provide a suitable measure to track this process.

**Electronic supplementary material:**

The online version of this article (10.1186/s13148-019-0617-1) contains supplementary material, which is available to authorized users.

## Background

Several protocols have been described for directed differentiation of iPSCs toward hematopoietic progenitor cells (iHPCs) [1–3]. These differentiation protocols frequently utilize a step-wise exposure of cells to specific cytokine cocktails that mimic multiple steps of mesoderm commitment and hematopoietic commitment [3–5]. iHPCs have been shown to express hematopoietic markers and to have a multipotent differentiation potential toward various hematopoietic lineages [6–9]. However, the efficiency of differentiation in terms of cell counts of resulting hematopoietic cells is rather low. More importantly, so far, iHPCs were not able to show long-term engraftment and thus iHPCs cannot be used in regenerative medicine [1, 5]. A better understanding of the molecular makeup of iHPCs is therefore important to further adjust differentiation procedures to ultimately achieve fully functional cells.

DNA methylation (DNAm) changes at CG dinucleotides (CpG sites) resemble an important epigenetic modification, which is relevant for normal hematopoietic differentiation into specific lineages [10, 11]. Conversely, DNAm patterns can be utilized to characterize the cellular composition of blood [12, 13]. Recently, Nishizawa et al. compared DNAm patterns among iPSC-lines that revealed varying differentiation potential toward the hematopoietic lineage depending on IGF2 levels in pluripotent cells [14]. This study also described DNAm profiles of iPSCs and iPSC-derived HPCs. However, a systematic comparison of DNAm profiles of iHPCs with normal hematopoietic counterparts was not yet performed.

It is well known that primary HPCs are regulated by tight interaction with their niche, which can be mimicked by co-culture with suitable feeder layers in vitro [15]. Mesenchymal stromal cells (MSCs) were shown to support culture expansion of primary HPCs [16–18], and it has even been tested in clinical trials if co-culture of HPCs with MSCs increases hematopoietic engraftment in allogeneic transplantation [19]. Hence, it might be anticipated that co-culture of iHPCs with MSCs might further support culture expansion and hematopoietic commitment of iHPCs. MSC-like cells can also be derived from iPSCs [20–22], and we have previously described that such iMSCs support culture expansion of primary HPCs [23]. Thus, iPSCs might be differentiated in parallel toward either the hematopoietic or mesenchymal stromal cell lineage for subsequent co-culture in a syngenic setting. This might provide new perspectives to further increase differentiation efficiency.

In this study, we compared DNAm patterns of iHPCs with various hematopoietic cell types. We demonstrate that in particular patterns of the monocytic lineage are recapitulated, while DNAm patterns remained overall different from primary hematopoietic cells. There was no evidence that additional co-culture with syngenic MSCs or iMSCs supported further epigenetic maturation of iHPCs.

## Results

### Differentiation of iPSCs toward the hematopoietic lineage is reflected by corresponding DNA methylation changes

**Fig. 1:**
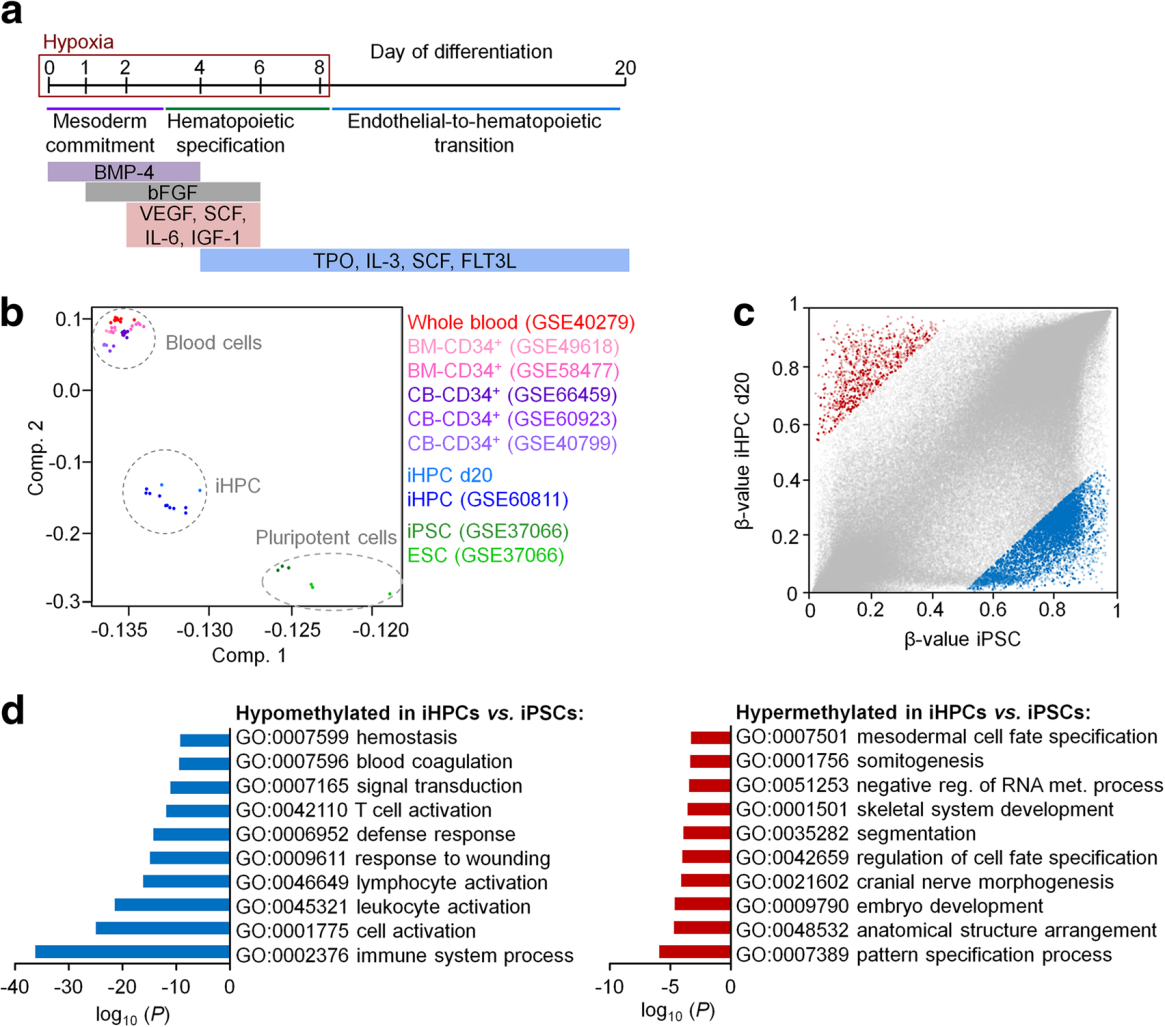
DNA methylation profiles of iPSC-derived hematopoietic progenitor cells. **a** Schematic representation of the differentiation protocol of iPSCs toward hematopoietic progenitor cells. **b** Principal component analysis of DNA methylation profiles of our iHPCs (donor 1 and 2) and those of Nishizawa et al. [14], primary CD34^+^ cells from cord blood (CB) or bone marrow (BM), whole blood, and pluripotent cells. **c** Scatter plot depicts CpG sites that are either hypermethylated (961 CpG sites, red) or hypomethylated (6075 CpG sites, blue; delta mean *β* value > 0.5 or < − 0.5) in iHPCs as compared to iPSC (GSE37066). CpG sites associated with promoter regions are highlighted in bold. **d** Gene ontology analysis of genes with differentially methylated CpG sites in the promoter region. Enrichment of specific categories was calculated by the one-sided Fisher’s exact *P* value

Directed differentiation of iPSCs into iHPCs was induced by embryoid body (EB) formation and subsequent stage-specific application of specific cytokine cocktails, as indicated in Fig. 1a [4, 24]. These experiments were done with three iPSC lines that were reprogrammed from MSCs of different donors. After 10 days, cells with typical hematopoietic morphology started to bud off from the endothelial layer and these cells were harvested from the supernatant after 20 days (Additional file 1: Fig. S1a). Cytospins revealed predominantly a rather monocytic morphology (Additional file 1: Fig. S1b). Immunophenotype analysis demonstrated high expression of CD31 and CD45, which is in line with hematopoietic cell fate decisions (Additional file 1: Fig. S1c, d). However, high expression of CD34, which is indicative for hematopoietic progenitor cells, was only observed on a subpopulation at day 6 and was downregulated at day 20. Similar findings were described by other authors before [4], and the results are overall in line with differentiation toward the hematopoietic lineage.

We have then analyzed DNAm profiles of two iPSC clones after 20 days of differentiation using the Illumina Infinium MethylationEPIC BeadChip. Principal component analysis demonstrated that iHPCs clustered closely together with iPSC-derived hematopoietic progenitor cells of Nishizawa et al. [14] (Fig. 1b). These authors used a differentiation protocol with a different cytokine composition and without hypoxic conditions. Thus, the epigenetic state of iHPCs appears to be independent of the differentiation regimen used. Notably, the iHPCs were overall clearly separated from primary hematopoietic cells, indicating that hematopoietic differentiation was epigenetically incomplete.

To better understand the epigenetic changes that are acquired during differentiation of iPSCs into iHPCs, we filtered for CpGs with more than 50% change in DNAm levels: 961 CpGs were hypermethylated and 6075 CpGs were hypomethylated in iHPCs as compared to iPSCs (Fig. 1c). These DNAm changes were enriched in the gene body and intergenic regions, whereas they were less present in the promoter regions that may have more impact on gene expression (Additional file 2: Fig. S2a). Two hundred twenty hypermethylated and 1493 hypomethylated CpGs were associated with promoter regions (Additional file 3: Tab. S1). These differentially methylated CpG sites show similar methylation patterns in iHPCs as compared to cord blood-derived CD34^+^ cells (Additional file 4: Fig. S3a). Gene ontology analysis of the associated genes revealed enrichment of hypomethylation in categories of the immune system, blood cell activation, and hemostasis, whereas hypermethylation was rather related to development and cell fate (Fig. 1d).

**Fig. 2:**
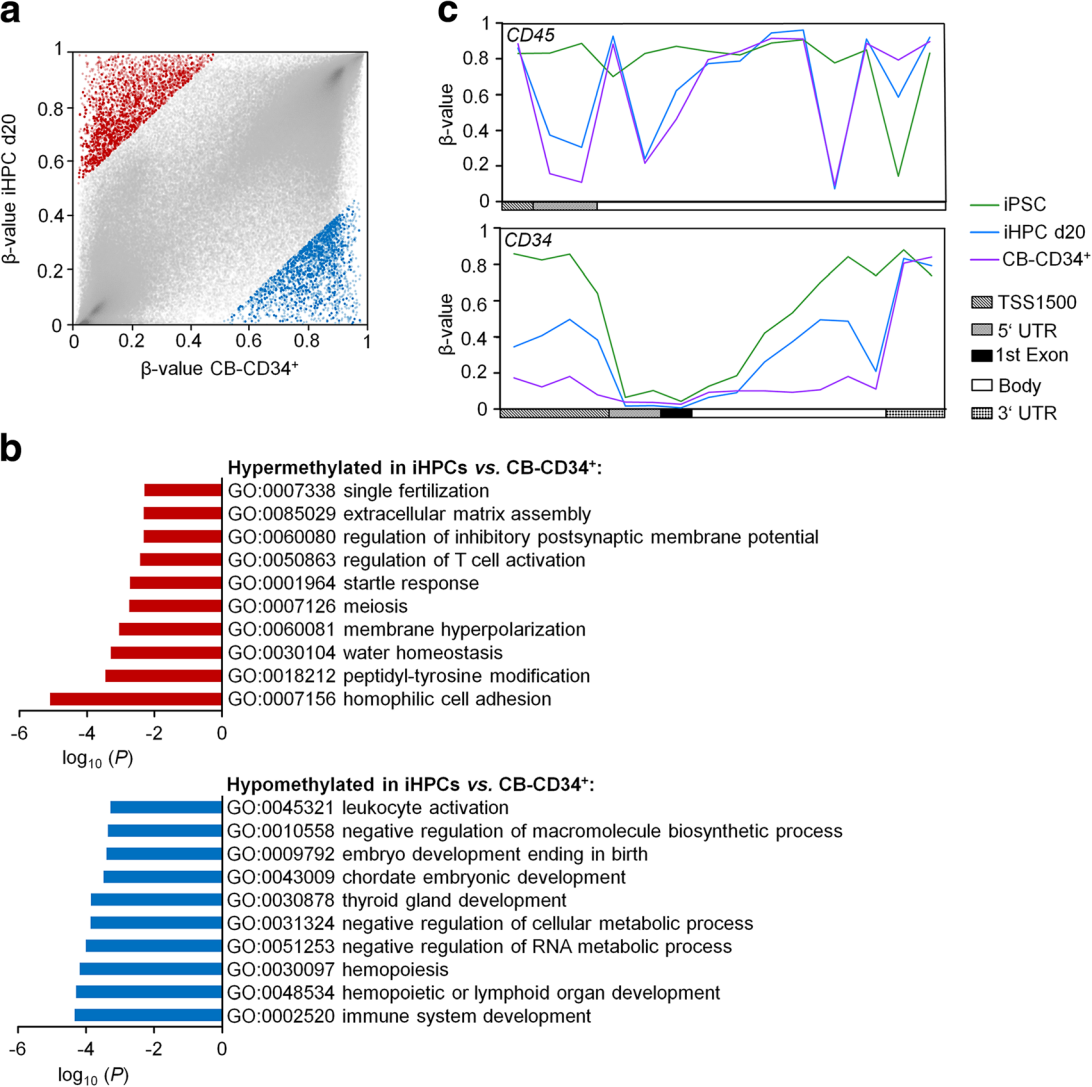
Comparison of DNA methylation profiles of iPSC-derived HPCs versus primary CD34^+^ cells. **a** Scatter plot of CpG sites that are either higher methylated (2692 CpG sites, red) or less methylated in iHPCs (2307 CpG sites, blue; delta mean *β* value > 0.5 or < − 0.5) compared to primary CD34^+^ cells. CpG sites associated with promoter regions are highlighted in bold (659 CpG sites and 587 CpG sites, respectively). **b** Gene ontology analysis of genes with differentially methylated CpG sites in the promoter region. Enrichment of specific categories was calculated by the one-sided Fisher’s exact *P* value. **c** DNA methylation patterns within the genes *CD45* and *CD34*. Mean DNAm levels are depicted for iPSCs (GSE37066), iHPC d20, and primary CD34^+^ cells. TSS1500: 1500 bp upstream of transcription start site; UTR: untranslated region. For all analyses, iHPCs were compared to primary CD34^+^ cells isolated from human cord blood (GSE40799)

Subsequently, we analyzed differential DNAm between iHPCs and primary CD34^+^ cells from umbilical cord blood (50% difference in DNAm levels): 2692 CpGs were higher methylated and 2307 CpGs were less methylated in iHPCs (Fig. 2a). Hypermethylation in iHPCs was enriched at intergenic regions, CpG islands, and their N shore region, whereas hypomethylation was enriched in 5′UTR, gene bodies, and N shore regions (Additional file 2: Fig. S2b). Six hundred fifty-nine hypermethylated and 587 hypomethylated CpGs were associated with promoter regions (Additional file 5: Tab. S2). Gene ontology analysis of the associated genes showed enrichment of hypermethylation in categories of various cellular processes, such as cell division or membrane potential, whereas hypomethylation was related to the immune system, hematopoiesis, development, and metabolic processes (Fig. 2b). These differentially regulated CpG sites do not recapitulate the DNAm patterns in MSCs but they were overall related to DNAm in iPSCs, indicating that these epigenetic modifications of hematopoietic development are not acquired in iHPCs during differentiation (Additional file 4: Fig. S3b). We have then exemplarily analyzed DNAm patterns within the genes *CD34* and *CD45*. Overall, iHPCs acquired a similar DNAm pattern as observed in primary CD34^+^ progenitor cells, particularly within the *CD45* gene (Fig. 2c). Thus, the DNAm profiles clearly recapitulated features of normal hematopoietic differentiation, albeit the overall epigenetic makeup was yet incomplete.

### Epigenetic changes point toward monocytic differentiation

**Fig. 3:**
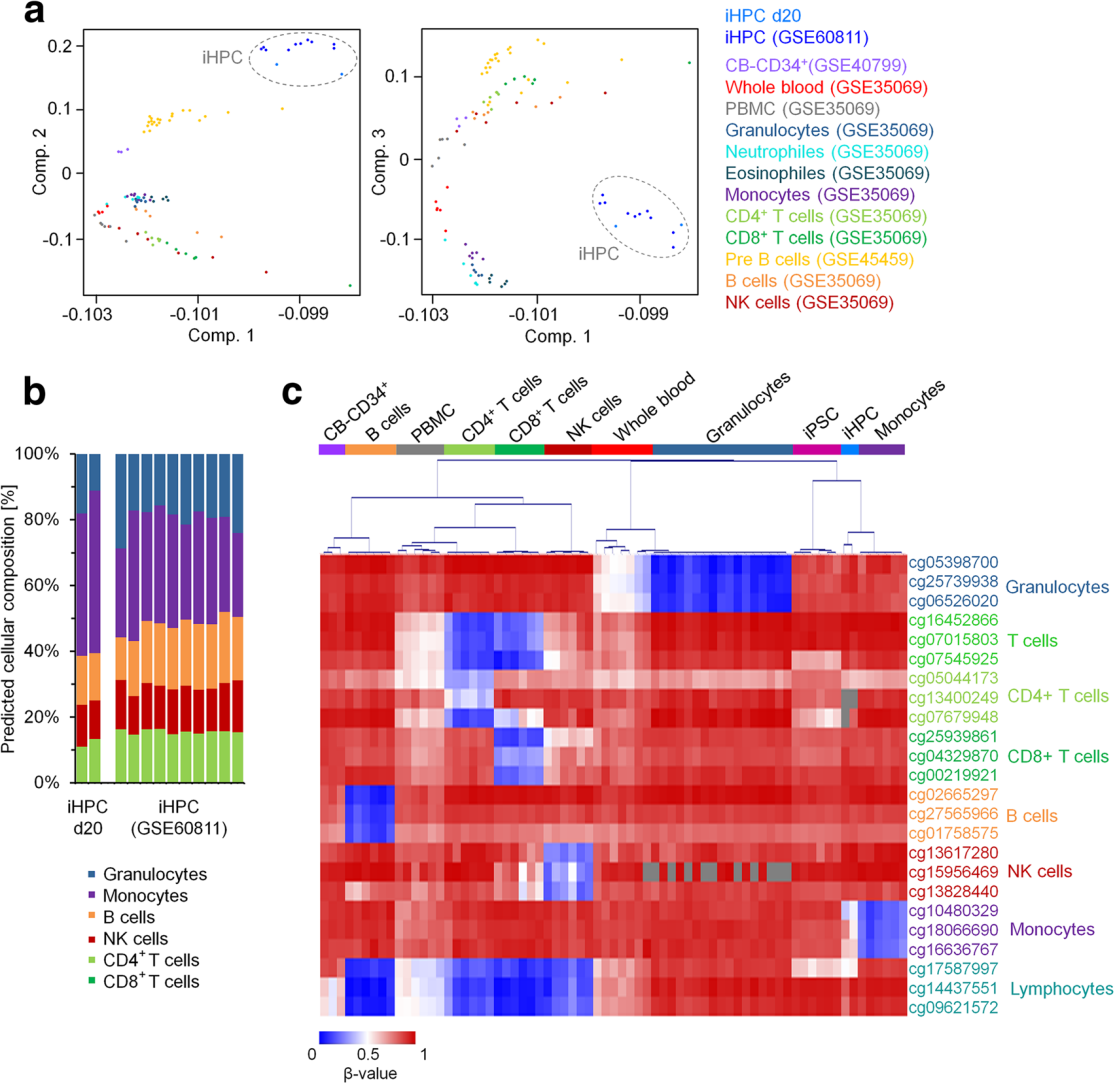
Comparison of DNA methylation profiles of iPSC-derived HPCs with different hematopoietic cell types. **a** Principal component analysis demonstrates that DNAm profiles of iHPCs are distinct to other cell types. **b** A deconvolution algorithm of Houseman et al. [13] was used to estimate the composition of different hematopoietic cell types based on the DNAm profiles of our iHPCs (donor 1 and 2) and those of Nishizawa et al. [14]. **c** Heatmap of DNAm levels at CpGs that are specifically hypomethylated in specific hematopoietic cell types. iHPCs (d20) are most closely related to monocytes (CD34^+^ cells isolated from human cord blood: GSE40799; B cells, peripheral blood mononuclear cells (PBMC), CD4^+^ T cells, CD8^+^ T cells, NK cells, whole blood, granulocytes, monocytes: all GSE35069; and iPSCs: GSE37066). Gray areas indicate that no data is available for this specific CpG site. CB: cord blood

We subsequently analyzed whether lineage-specific DNAm patterns are recapitulated in iHPCs. Integrative PCA analysis of DNAm profiles of iHPCs and various hematopoietic cell types demonstrated that the epigenetic profiles of iHPCs were rather distinct from all other cell types (Fig. 3a). We then used a deconvolution algorithm that has been developed to estimate the percentages of cell types in blood based on DNAm profiles [13]. The results indicated that the majority of our iHPCs and of the iPSC-derived progenitor cells generated by Nishizawa et al. [14] were classified as monocytes (Fig. 3b). However, this tool has been developed for primary cells and only includes few different cell types. Therefore, we independently filtered for specific sets of CpGs that are characteristically hypomethylated in granulocytes, CD4^+^ T cells, CD8^+^ T cells, B cells, NK cells, monocytes, or lymphocytes, as described before [12]. Clustering according to DNAm levels in these cell type-specific CpGs supported the notion that iHPCs recapitulate DNAm patterns of the monocytic lineage (Fig. 3c).

### Mesenchymal stromal cells support proliferation of iPSC-derived hematopoietic progenitor cells

**Fig. 4:**
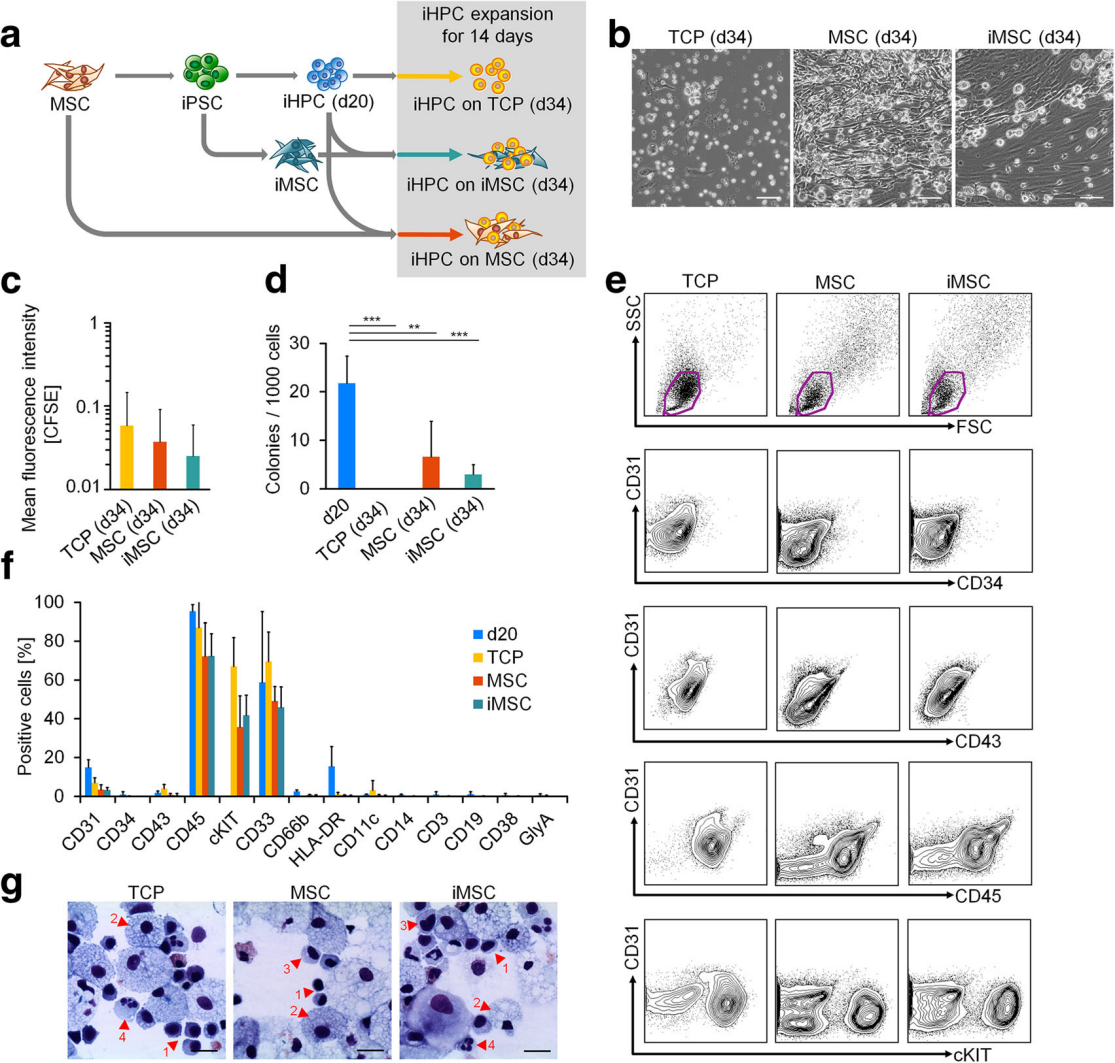
Co-culture with MSCs or iPSC-derived MSCs supports growth of iPSC-derived HPCs. **a** Schematic representation of co-culture experiments of iHPCs with syngenic MSCs and iMSCs (donors 1–3). **b** Phase contrast images of iHPCs upon expansion for 2 weeks on tissue culture plastic (TCP), MSCs, and iMSCs. Scale bar 100 μm. **c** Proliferation of iHPCs on TCP, MSCs, or iMSCs was estimated by residual CFSE stain after 5 days. The mean fluorescence intensities (MFI) were normalized to corresponding measurements at d0. Data represent the mean of three independent experiments ± SD (ANOVA was not significant). **d** Colony-forming unit (CFU) frequency of iHPCs at day 20 and after 14 days of co-culture expansion on TCP, MSCs, or iMSCs. Data represent mean of three independent experiments ± SD; ANOVA: **P < 0.01; ***P < 0.001. **e** Flow cytometry analysis of iHPCs on day 14 of co-culture as depicted in **a**. **f** Quantitative analysis of flow cytometric measurements. GlyA: glycophorin A. **g** Cytospins of iHPCs at day 34. Morphologies relate to hematopoietic progenitor cells (1), macrophages (2), monocytes (3), and neutrophils (4). Scale bar 20 μm

Subsequently, we followed the hypothesis that co-culture with MSCs might provide a cellular microenvironment that stimulates further differentiation toward the hematopoietic lineage. To this end, we used MSCs and iPSC-derived MSCs (iMSCs) that were generated from the same donors used for the reprogramming of iPSCs and differentiation into iHPCs (Fig. 4a). iMSCs revealed typical MSC-like morphology, immunophenotype, and three-lineage differentiation potential (Additional file 6: Fig. S4). Upon culture expansion for an additional 14 days on MSC and iMSC stroma or tissue culture plastic (TCP) as control, the vast majority of cells were non-adherent round cells with typical hematopoietic morphology (Fig. 4b). Proliferation of iHPCs was faster under co-culture conditions (Fig. 4c), which is in line with the accelerated proliferation of primary HPCs on a suitable feeder layer [16]. Furthermore, colony-forming unit (CFU) potential was maintained under co-culture conditions with MSCs and iMSCs, but not on TCP (Fig. 4d). However, compared to the iHPCs at day 20 without additional culture expansion, the proportion of cells with CFU forming potential was significantly decreased upon culture expansion. Flow cytometric analysis demonstrated that hematopoietic markers except for cKIT were downregulated by additional culture expansion (Fig. 4e,f). Cellular morphology in cytospins indicated multilineage hematopoietic differentiation, albeit most iHPCs were relatively large with a foam-like appearance of the cytoplasm that might be related to a macrophage phenotype [24] (Fig. 4g). Thus, the stromal support of MSCs or iMSCs does not seem to improve maintenance of hematopoietic progenitors and their multilineage potential as compared to iHPCs without further culture expansion.

### Additional culture expansion does not result in epigenetic maturation

**Fig. 5:**
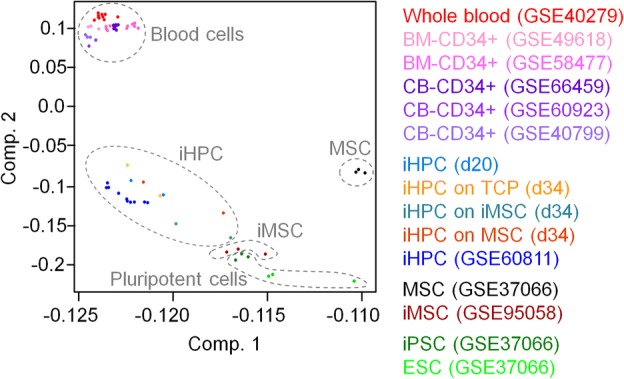
Principal component analysis of DNA methylation profiles of iPSC-derived HPCs with and without co-culture. The DNA methylation profiles of iHPCs (d20; donor 1 and 2) and iPSC-derived HPCs from Nishizawa et al. [14] are closely related to those of iHPCs that were further cultured for 2 weeks with or without stromal support. Additional stromal support (iHPC on MSCs, iMSCs, and TCP, respectively) did not enhance further commitment toward the hematopoietic lineage

To estimate the impact of stromal support on the epigenetic makeup of iHPCs, we analyzed DNAm profiles of the expanded cell preparations. Albeit co-cultures resembled a mixture of iHPCs with MSCs or iMSCs, we did not separate the cell types by cell sorting, as the results would be largely affected by sorting strategies and the majority of cells seemed to be iHPC-derived. In fact, PCA demonstrated that the additional culture expansion for 2 weeks with or without stromal support resulted in DNAm profiles that remained similar to those of iHPCs at day 20 (Fig. 5). In tendency, co-cultured iHPCs clustered closer to iMSCs than the non-co-cultured iHPCs due to the residual feeder cells. Either way, the results demonstrated that additional culture expansion of iHPCs either with or without stromal support did not support further epigenetic maturation toward a DNAm profile of the hematopoietic lineage.

## Discussion

Generation of hematopoietic progenitor cells from iPSCs opens new perspectives for patient-specific disease modeling and drug testing. Furthermore, it may eventually overcome limitations in culture expansion of primary HPCs for cellular therapy. However, the results of this study support the notion that with current differentiation regimen, iHPCs remain epigenetically distinct from their in vivo counterparts.

Optimization of differentiation conditions for iHPCs remains a challenge [3]. Most protocols seem to be biased toward myeloid differentiation, which is in line with results shown here demonstrating that DNAm profiles are related to the monocytic lineage. The immunophenotype of our iHPCs is overall similar to previous studies, albeit, for example, Sontag et al. [24] and Dorn et al. [25] described a more pronounced upregulation of CD43. More recently, several groups suggested alternative protocols that utilize overexpression of specific transcription factors or activation of signal cascades that may better support definitive hematopoiesis resulting in improved engraftment capabilities of hematopoietic progenitors [5, 26, 27]. In the future, it will be interesting to investigate if these approaches give rise to progeny that resemble their primary counterparts more closely on epigenetic levels.

DNA methylation has been demonstrated to be an important regulative mechanism for cell-fate decisions in hematopoietic differentiation [11, 28]. Here, we show that DNAm profiles of our iHPCs are closely related to those by Nishizawa and colleagues [14], although these authors did not use the same differentiation conditions. It will be important to gain further understanding of how epigenetic profiles of iPSC-derived hematopoietic cells are affected by various differentiation regimens. The discrepancy in the epigenetic makeup of iHPCs versus in vivo hematopoietic stem and progenitor cells may at least be partially attributed to other epigenetic signatures that are not directly related to lineage-decisions, but rather to attributes such as tissue-specific and age-associated DNAm patterns [20, 29].

It has been shown that early steps in normal hematopoietic development are reflected by continuous gain or loss of DNAm during differentiation and that these changes are overall inversely correlated with gene expression at key loci [28, 30]. This is in line with our observation that demethylation in iHPCs is particularly enriched in promoter regions of genes that are relevant for hematopoietic differentiation. On the other hand, association of DNAm with gene expression is frequently rather low [28, 31, 32]. It may be too simplistic to consider only DNAm changes as functionally relevant that directly impact on gene expression changes. DNAm patterns are also closely related to other epigenetic marks, such as histone modifications and higher order chromatin conformation. These aspects should be taken into consideration for future studies.

Mesenchymal stromal cells represent an important component of the hematopoietic stem cell niche [33]. They secrete various chemokines and facilitate cell-cell interactions that were shown to support primary HPCs also in vitro [16, 34]. In this study, we demonstrate that MSCs and iMSCs support proliferation and maintenance of CFU potential of iHPCs. However, our results do not indicate that this additional co-culture regimen increases CFU frequency or maturation of a hematopoietic immunophenotype. Furthermore, the additional culture expansion, either with or without stromal support, does not result in epigenetic maturation of iHPCs to their in vivo counterparts. It is conceivable that co-culture needs to be implemented at earlier steps of the differentiation procedure or that it needs to be combined with other culture media.

## Conclusion

Differentiation of iPSC toward the hematopoietic lineage is associated with DNAm profiles that point toward normal hematopoietic differentiation, but the epigenetic patterns remain incomplete. It will be important to investigate whether epigenetic assimilation can be improved by alternative regimen for hematopoietic differentiation, and for this purpose, DNAm profiles provide an important measure for evaluation of differentiation procedures.

## Methods

### Cell culture

Mesenchymal stromal cells were isolated from bone marrow (*caput femoris*) of patients undergoing orthopedic surgery and culture-expanded in standard medium consisting of Dulbecco’s Modified Eagle Medium (DMEM; 1 g/l glucose; PAA, Pasching, Austria), 1% L-glutamine (PAA), 1% penicillin/streptomycin (PAA), and 10% pooled human platelet lysate (hPL) [35]. The medium was supplemented with 0.1% heparin (5000 IU/ml; Ratiopharm, Ulm, Germany) to prevent coagulation [36].

Induced pluripotent stem cells were generated from three MSC preparations with episomal plasmids [37] and thoroughly characterized as described before [38, 39]. iPSCs were cultured on tissue culture plastic (TCP) coated with vitronectin (0.5 mg/cm^2^) in StemMACS iPS-Brew XF (all Miltenyi Biotec, Bergisch Gladbach, Germany). Pluripotency was validated by in vitro differentiation and Epi-Pluri-Score (Cygenia GmbH, Aachen, Germany) [40].

Generation of iPSC-derived MSCs (iMSCs) was performed by switching culture conditions to standard hPL-medium and further passaging on 0.1% gelatin-coated plates [20]. Three-lineage differentiation potential of MSCs and iMSCs was validated as described before [41, 42]. Cell images were taken on a digital EVOS FL Auto microscope (Thermo Fisher Scientific, Carlsbad, California, USA).

### Hematopoietic progenitor differentiation

Differentiation of iPSCs toward hematopoietic progenitor cells was performed as described before with iPSCs from three different donors [24]. In brief, iPSC colonies were harvested and aggregated into embryoid bodies (EBs), which were cultured in basal differentiation medium consisting of StemPro34 supplemented with 100 U/ml penicillin, 100 μg/ml streptomycin, 2 mM L-glutamine (all Thermo Fisher Scientific), 0.4 mM monothioglycerol, and 50 μg/ml L-ascorbic acid (all Sigma Aldrich, St. Louis, Missouri, USA). The following growth factors were added as indicated in Fig. 1a: 8 ng/ml bone morphogenic protein 4 (BMP4), 10 ng/ml human basic fibroblast growth factor (bFGF), 10 ng/ml vascular endothelial growth factor (VEGF), 100 ng/ml interleukin 6 (IL-6), 100 ng/ml stem cell factor (SCF), 25 ng/ml insulin-like growth factor 1 (IGF1), 30 ng/ml interleukin 3 (IL-3), 20 ng/ml thrombopoietin (TPO), and 10 ng/ml fms-related tyrosine kinase 3 ligand (FLT3L). BMP4, IL-3, IL-6, and SCF were purchased from Miltenyi Biotec, and all other cytokines from Peprotech (Hamburg, Germany). Between day 0 and day 7, cells were cultured in a 5% O_2_ and 5% CO_2_ atmosphere. On day 6, cells were moved to 0.1% gelatin-coated TCP, and on day 8, cultures were transferred to normoxia and 5% CO_2_ atmosphere. On day 20, iHPCs were harvested for analysis or additional co-culture by collecting the supernatant, washing the well twice with PBS and centrifugation of the cells.

### Immunophenotypic analysis

Flow cytometric analysis of hematopoietic surface markers was analyzed on a FACS Canto II (BD Biosciences, Franklin Lakes, New Jersey, USA) with the following antibodies: CD3-PerCP-Cy5.5 (clone UCHT1), CD19-FITC (clone HIB19), CD31-PE (clone WM59), CD34-APC (clone 581), CD38-PE (clone HIT2), CD43-FITC (clone 1G10), CD14-PE (clone MOP9), CD66b-PE (clone G10F5) (all BD Bioscience), CD45-APC-Cy7 (clone 2D1), CD117-PE-Cy7 (clone 104D2), CD11c-PE-Cy7 (clone 3.9), HLA-DR-FITC (clone LN3), CD235a-Pacific Blue (clone 6A7M) (all eBioscience, San Diego, California, USA), CD33-APC (clone AC104.3E3; Miltenyi Biotec). EBs were dissociated with Accutase (Stemcell Technologies, Vancouver, British Columbia, Canada) for 10–15 min prior to staining with antibodies. Single cells were incubated with 1% human IgG solution (Privigen, CSL Behring, King of Prussia, Pennsylvania, USA) for 30 min at 4 °C to block unspecific binding.

Immunophenotypic analysis of MSCs and iMSCs was performed with CD14-APC (clone M5E2), CD29-PE (clone MAR4), CD31-PE (clone WM59), CD34-APC (clone 581), CD45-APC (clone HI30), CD73-PE (clone AD2), CD90-APC (clone 5E10) (all BD Biosciences), and CD105-FITC (clone MEM-226; ImmunoTools, Friesoythe, Germany). Data was analyzed using the FlowJo software (Tree Star, Ashland, Oregon, USA).

### Cytospin

Cellular morphology of iHPCs was analyzed by cytospins with Diff Quik staining (Medion Grifols Diagnostics, Düdingen, Switzerland) and analyzed with a Leica DMRX microscope (Leica Microsystems, Wetzlar, Germany).

### Colony-forming unit assay

After 20 days of differentiation or after additional co-culture with MSCs or iMSCs for 2 weeks (day 34 of differentiation), 1000 iHPCs were seeded in 1 ml of methylcellulose-based medium (HSC-CFU lite with EPO; Miltenyi Biotec) in 24-well plates. Colonies were quantified 14 days later.

### Proliferation analysis

Cells were labeled with Carboxyfluorescein N-succinimidyl ester (CFSE; Sigma-Aldrich) and the residual dye was analyzed after 5 days by flow cytometry, as described before [16].

### Co-culture conditions

Mesenchymal stromal cells, iPSCs, and iMSCs were generated from the same three donors. After culture expansion, MSCs and iMSCs were irradiated (30 Gy) and used as feeder layers at a density of 50,000 cells/cm^2^ on 0.1% gelatin-coated dishes. After differentiation, 100,000 iHPCs were seeded onto MSCs, iMSCs, or TCP in StemSpan SFEM (Stemcell Technologies, Cologne, Germany) supplemented with 10 ng/ml fibroblast growth factor 1 (FGF-1), 10 ng/ml SCF, 10 ng/ml TPO (all purchased from Peprotech), and 1% penicillin/streptomycin [15].

### DNA methylation analysis

Genomic DNA of iHPCs from two donors was harvested with the NucleoSpin Tissue kit (Macherey-Nagel, Düren, Germany), bisulfite-converted with the EZ DNA Methylation™ Kit (Zymo Research, Irvine, California, USA), and analyzed on the Infinium MethylationEPIC BeadChip (Illumina, San Diego, California, USA). Hybridization and initial data processing with the GenomeStudio (v2011.1) Methylation Module (v1.9.0) was performed at Life&Brain (Bonn, Germany). Raw data has been deposited at Gene Expression Omnibus (GEO) under the accession number GSE119079. In addition, we used previously published DNAm profiles of iHPCs (GSE60811) [14]; MSCs, embryonic stem cells (ESCs), and iPSCs (all GSE37066) [43]; iMSCs (GSE95058) [38]; cord blood-derived and bone marrow-derived CD34^+^ cells (GSE40799 [44], GSE60923 [14], GSE66459 [45], GSE49618 [46], GSE58477 [47]); whole blood (GSE40279 [48], GSE35069 [49]); and different hematopoietic subsets (GSE35069 [49], GSE45459 [50]).

For further analysis, we focused on CpGs that were represented on both the Infinium HumanMethylation450K BeadChip (in total 485,577 CpGs on the platform) and the Infinium MethylationEPIC BeadChip (in total 867,926 CpGs) with an overlap of 454,181 CpG sites on both platforms. CpGs on X and Y chromosomes were excluded resulting in 443,542 CpGs for further analysis. More than 99% of these filtered CpGs were detected in each iHPC sample. Principal component analysis (PCA) was performed with R; heatmaps and hierarchical clustering (HCL) were generated with the MultiExperiment Viewer (MeV; version 4.9.0). Differentially methylated CpGs were selected by the mean difference in DNAm as indicated in the text. For enrichment analysis, CpG sites were classified according to gene regions and CpG islands as described before [43, 51]. Gene ontology (GO) analysis was performed on genes with differentially methylated CpGs in the promoter region (located in TSS1500, TSS200, and 5′UTR) using the GoMiner software [52]. Categories comprising more than 1000 genes were not considered and similar categories are only listed once.

### Statistical analysis

Proliferation, immunophenotypic, and CFU experiments were performed with three independent biological replicates and results are presented as mean ± standard deviation (SD). Statistical significance was estimated by unpaired one-way ANOVA in GraphPad Prism 6. Enrichment of gene ontology categories was calculated by the one-sided Fisher’s exact *P* value using all genes represented on the array as a reference (within the 443,542 CpGs that were filtered for this analysis as described above). For enrichment analyses in gene regions or CpG islands, statistical significance was estimated by hypergeometric distribution in Microsoft Excel.

## Additional files

Additional file 1: **Figure S1.** Differentiation of iPSCs toward hematopoietic progenitor cells. (a) Phase contrast images in the course of hematopoietic differentiation cultures (on days 0, 6, 10, and 20). Scale bar = 500 μm and scale bar in inlet = 100 μm. (b) Exemplary cytospin analysis on day 22 reveals prevailing monocytic morphology. Scale bar = 10 μm. (c) Flow cytometry analysis of hematopoietic differentiation cultures on days 6 and 20. Blots are representative for three independent experiments. (d) Frequencies of CD31, CD34, CD43, CD45, and cKIT populations on day 6 and 20 of differentiation. Data represent the mean of three independent experiments ± SD. (PDF 376 kb)

Additional file 2: **Figure S2.** Enrichment of hyper- and hypomethylated CpG sites in iPSC-derived HPCs. Changes in DNA methylation (delta mean *β* value > 0.5 or < − 0.5) in comparison (a) between iHPCs d20 and iPSCs (GSE37066) or (b) between iHPCs d20 and primary CD34^+^ cells from human cord blood (GSE40799). The differentially methylated CpGs were classified according to gene regions and in relation to CpG islands. Hypergeometric distribution: *P < 0.05; ^#^*P* < 0.01; ^$^*P* < 0.001; ^§^*P* < 0.0001; ^♦^*P* < 0.00001. (PDF 344 kb)

Additional file 3: **Table S1.** Differentially methylated CpGs in iPSC-derived HPCs versus iPSCs. Promoter-associated CpG sites that are either hypermethylated (220 CpG sites) or hypomethylated (1493 CpG sites; delta mean *β* value > 0.5 or < − 0.5) in iHPCs d20 compared to iPSCs (GSE37066) with related genes, gene groups, association to CpG islands, and mean *β* values of the cell types. (XLSX 119 kb)

Additional file 4: **Figure S3.** Comparison of differentially methylated CpG sites across different cell types. Heatmap of DNAm levels at promoter-associated CpG sites that are either at least 50% hypo- or hypermethylated in (a) iHPCs versus iPSCs (corresponding to Fig. 1c) or in (b) iHPCs versus cord blood-derived CD34^+^ cells (corresponding to Fig. 2a). DNAm levels are compared between MSCs, iPSCs, iHPCs d20, and cord blood-derived CD34^+^ cells. The heatmaps were sorted by the mean DNAm levels in MSCs. (PDF 126 kb)

Additional file 5: **Table S2.** Differentially methylated CpGs in iPSC-derived HPCs versus CD34^+^ cells. Promoter-associated CpG sites that are either hypermethylated (659 CpG sites) or hypomethylated (587 CpG sites; delta mean *β* value > 0.5 or < − 0.5) in iHPCs compared to human cord blood-derived CD34^+^ cells (GSE40799) with related genes, gene groups, association to CpG islands, and mean *β* values of the cell types. (XLSX 91 kb)

Additional file 6: **Figure S4.** Differentiation of iPSCs toward MSCs. (a) Phase contrast images of iPSCs and in the course of differentiation toward iPSC-derived MSCs on day 5, 10, 20, and 30. Scale bar = 100 μm. (b) Flow cytometric analysis of iMSCs, MSCs, and iPSCs. Data is representative of three independent experiments. Autofluorescence is indicated in white. (c) iMSCs can be differentiated into adipocytes (BODIPY staining of fat droplets), osteocytes (Alizarin Red staining) and chondrocytes (Alcian Blue/PAS staining). (PDF 342 kb)

## Abbreviations

CB: Cord blood
CFU: Colony-forming unit
DNAm: DNA methylation
ESC: Embryonic stem cell
HPC: Hematopoietic progenitor cell
iHPC: Induced pluripotent stem cell-derived hematopoietic progenitor cell
iMSC: Induced pluripotent stem cell-derived mesenchymal stromal cell
iPSC: Induced pluripotent stem cell
MSC: Mesenchymal stromal cell
PCA: Principal component analysis
TCP: Tissue culture plastic
TSS: Transcription start site
UTR: Untranslated region
