# Supplementary material for: Tracking of epigenetic changes during hematopoietic differentiation of induced pluripotent stem cells
Source: Clin Epigenetics. 2019 Feb 4;11:19. doi: 10.1186/s13148-019-0617-1 (PMC6360658; doi:10.1186/s13148-019-0617-1)
Supplement: Supplementary file 1 — Figure S1. Differentiation of iPSCs toward hematopoietic progenitor cells. (a) Phase contrast images in the course of hematopoietic differentiation cultures (on days 0, 6, 10, and 20). Scale bar = 500 μm and scale bar in inlet = 100 μm. (b) Exemplary cytospin analysis on day 22 reveals prevailing monocytic morphology. Scale bar = 10 μm. (c) Flow cytometry analysis of hematopoietic differentiation cultures on days 6 and 20. Blots are representative for three independent experiments. (d) Frequencies of CD31, CD34, CD43, CD45, and cKIT populations on day 6 and 20 of differentiation. Data represent the mean of three independent experiments ± SD. (PDF 376 kb) [file 13148_2019_617_MOESM1_ESM.pdf]

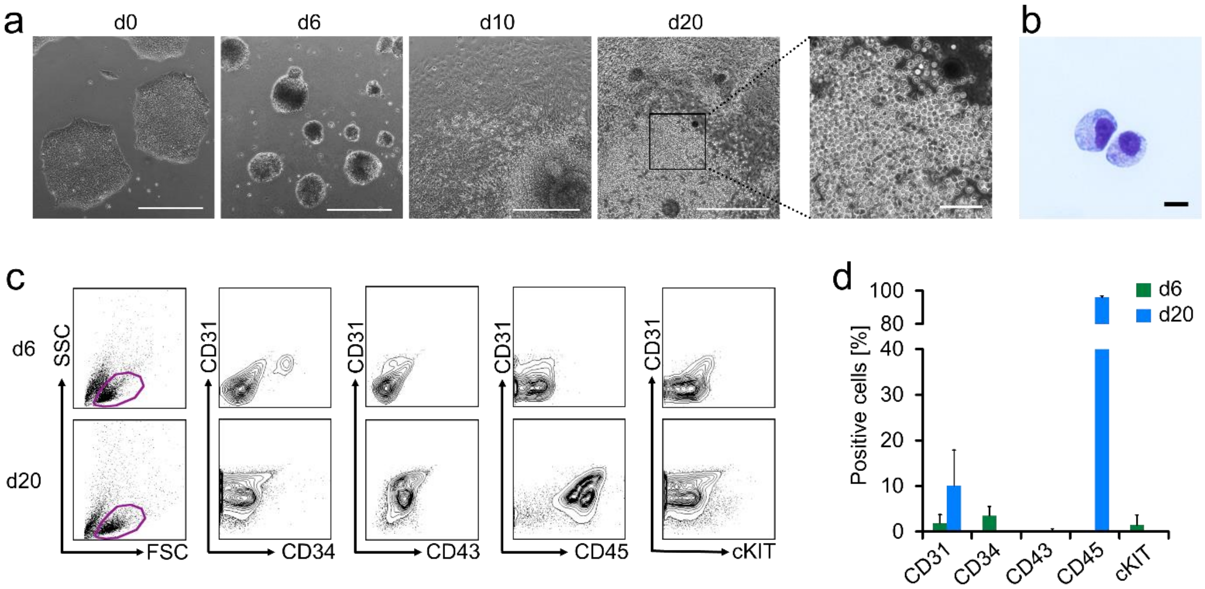

Fig. S1: Differentiation of iPSCs toward hematopoietic progenitor cells.

(a) Phase contrast images in the course of hematopoietic differentiation cultures (on days 0, 6, 10, and 20). Scale bar = 500 µm and scale bar in inlet = 100 µm. **(b)** Exemplary cytospin analysis on day 22 reveals prevailing monocytic morphology. Scale bar = 10 µm. **(c)** Flow cytometry analysis of hematopoietic differentiation cultures on days 6 and 20. Blots are representative for three independent experiments. **(d)** Frequencies of CD31, CD34, CD43, CD45, and cKIT populations on day 6 and 20 of differentiation. Data represent the mean of three independent experiments ± SD.
