# Supplementary material for: Tracking of epigenetic changes during hematopoietic differentiation of induced pluripotent stem cells
Source: Clin Epigenetics. 2019 Feb 4;11:19. doi: 10.1186/s13148-019-0617-1 (PMC6360658; doi:10.1186/s13148-019-0617-1)
Supplement: Supplementary file 2 — Figure S2. Enrichment of hyper- and hypomethylated CpG sites in iPSC-derived HPCs. Changes in DNA methylation (delta mean β value > 0.5 or < − 0.5) in comparison (a) between iHPCs d20 and iPSCs (GSE37066) or (b) between iHPCs d20 and primary CD34+ cells from human cord blood (GSE40799). The differentially methylated CpGs were classified according to gene regions and in relation to CpG islands. Hypergeometric distribution: *P < 0.05; #P < 0.01; $P < 0.001; §P < 0.0001; ♦P < 0.00001. (PDF 344 kb) [file 13148_2019_617_MOESM2_ESM.pdf]

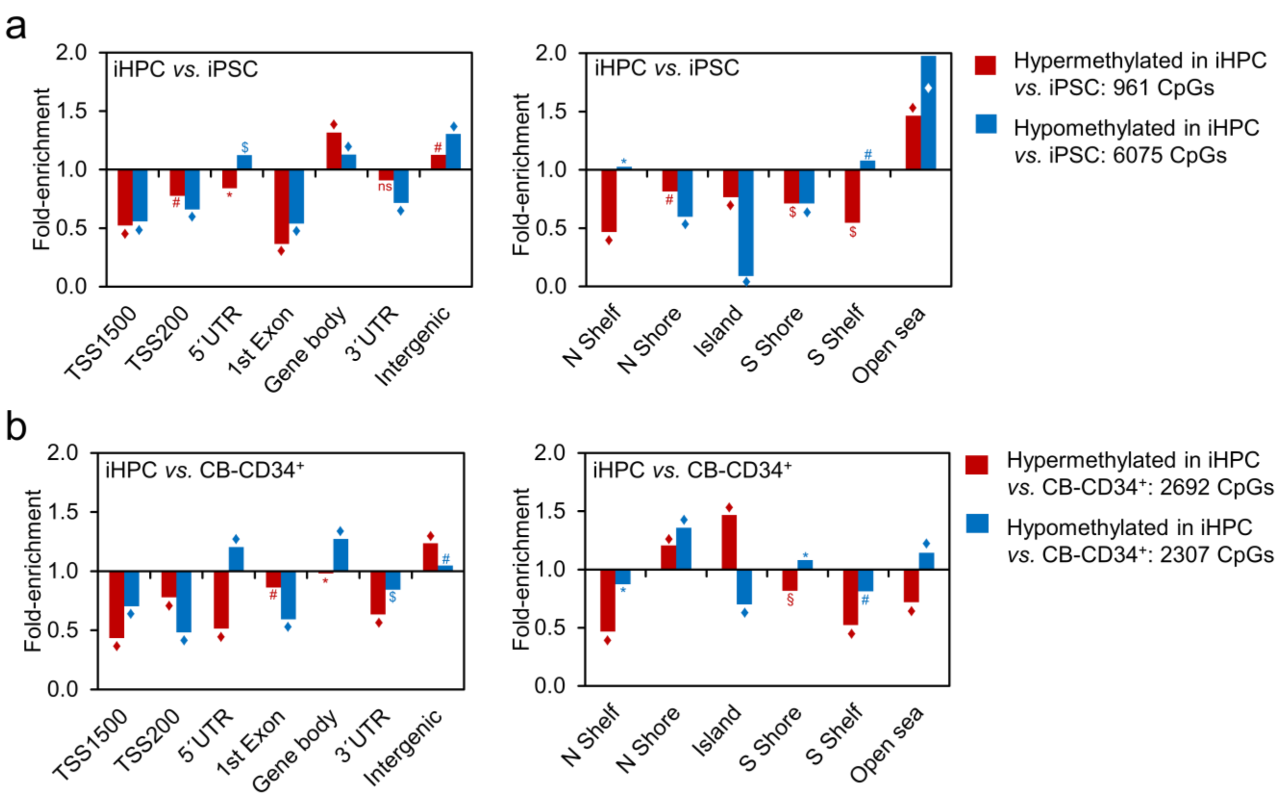

Fig. S2: Enrichment of hyper- and hypomethylated CpG sites in iPSC-derived HPCs. Changes in DNA methylation (delta mean β-value >0.5 or <-0.5) in comparison **(a)** between iHPCs d20 and iPSCs (GSE37066) or **(b)** between iHPCs d20 and primary $CD34^+$ cells from human cord blood (GSE40799). The differentially methylated CpGs were classified according to gene regions and in relation to CpG islands. Hypergeometric distribution: * = $P < 0.05$; # = $P < 0.01$; \$ = $P < 0.001$; § = $P < 0.0001$; ♦ = $P < 0.00001$.
