# Supplementary material for: Tracking of epigenetic changes during hematopoietic differentiation of induced pluripotent stem cells
Source: Clin Epigenetics. 2019 Feb 4;11:19. doi: 10.1186/s13148-019-0617-1 (PMC6360658; doi:10.1186/s13148-019-0617-1)
Supplement: Supplementary file 4 — Figure S3. Comparison of differentially methylated CpG sites across different cell types. Heatmap of DNAm levels at promoter-associated CpG sites that are either at least 50% hypo- or hypermethylated in (a) iHPCs versus iPSCs (corresponding to Fig. 1c) or in (b) iHPCs versus cord blood-derived CD34+ cells (corresponding to Fig. 2a). DNAm levels are compared between MSCs, iPSCs, iHPCs d20, and cord blood-derived CD34+ cells. The heatmaps were sorted by the mean DNAm levels in MSCs. (PDF 126 kb) [file 13148_2019_617_MOESM4_ESM.pdf]

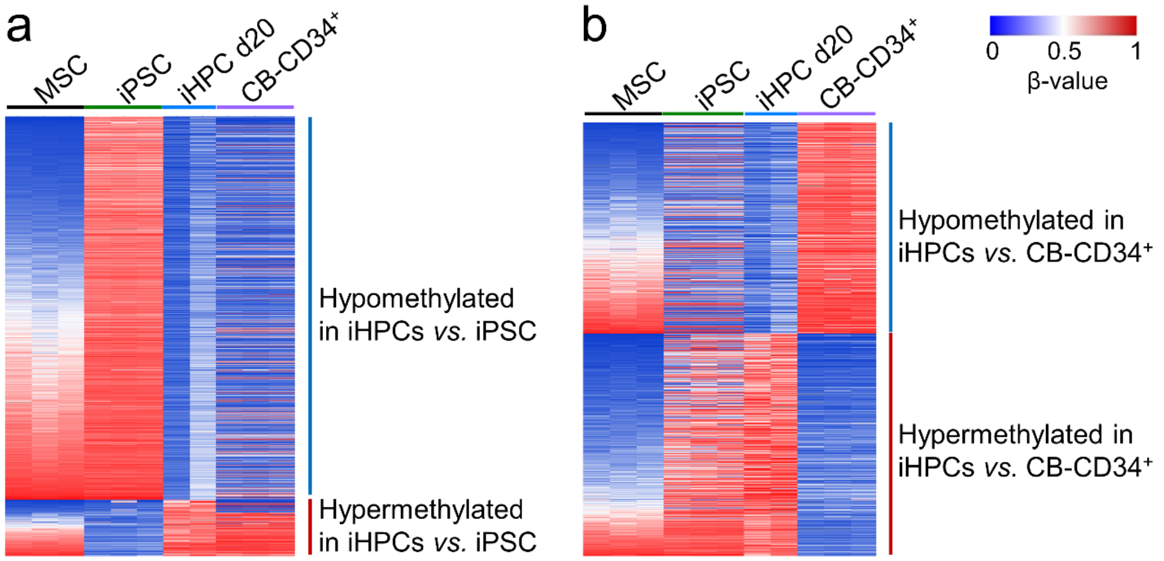

Fig. S3: Comparison of differentially methylated CpG sites across different cell types. Heatmap of DNAm levels at promoter associated CpG sites that are either 50 % hypo- or hypermethylated in **(a)** iHPCs *versus* iPSCs (corresponding to Figure 1C) or in **(b)** iHPCs *versus* cord blood-derived $CD34^+$ cells (corresponding to Figure 2A). DNAm levels are compared between MSCs, iPSCs, iHPCs d20, and cord blood-derived $CD34^+$ cells. The heatmaps were sorted by the mean DNAm levels in MSCs.
