# Supplementary material for: Tracking of epigenetic changes during hematopoietic differentiation of induced pluripotent stem cells
Source: Clin Epigenetics. 2019 Feb 4;11:19. doi: 10.1186/s13148-019-0617-1 (PMC6360658; doi:10.1186/s13148-019-0617-1)
Supplement: Supplementary file 6 — Figure S4. Differentiation of iPSCs toward MSCs. (a) Phase contrast images of iPSCs and in the course of differentiation toward iPSC-derived MSCs on day 5, 10, 20, and 30. Scale bar = 100 μm. (b) Flow cytometric analysis of iMSCs, MSCs, and iPSCs. Data is representative of three independent experiments. Autofluorescence is indicated in white. (c) iMSCs can be differentiated into adipocytes (BODIPY staining of fat droplets), osteocytes (Alizarin Red staining) and chondrocytes (Alcian Blue/PAS staining). (PDF 342 kb) [file 13148_2019_617_MOESM6_ESM.pdf]

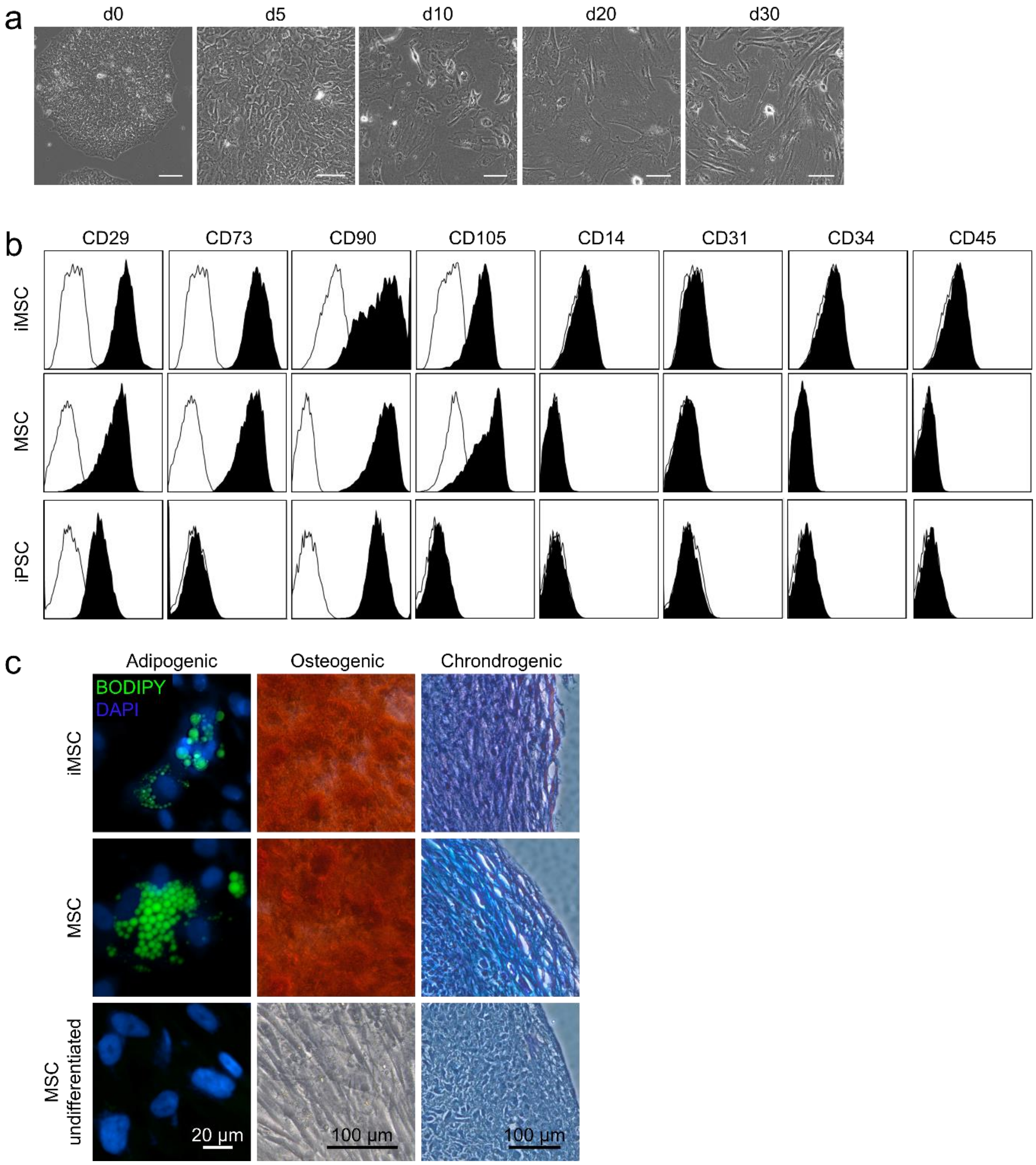

Fig. S4: Differentiation of iPSCs toward MSCs.

(a) Phase contrast images of iPSCs and in the course of differentiation toward iPSC-derived MSCs on day 5, 10, 20, and 30. Scale bar = 100 µm. **(b)** Flow cytometric analysis of iMSCs, MSCs, and iPSCs. Data is representative of three independent experiments. Autofluorescence is indicated in white. **(c)** iMSCs can be differentiated into adipocytes (BODIPY staining of fat droplets), osteocytes (Alizarin Red staining) and chondrocytes (Alcian Blue/PAS staining).
